# Transseptal Approach versus Left Atrial Approach to Mitral Valve: A Propensity Score Matching Study 

**Published:** 2015-10-27

**Authors:** Omid Rezahosseini, Mohamadreza Rezaei, Seyed Hossein Ahmadi Tafti, Arash Jalali, Payvand Bina, Atefeh Ghiasi, Abbasali Karimi, Kiomars Abbasi, Mahmood Shirzad, Saeed Davoodi, Abbas Salehi Omran

**Affiliations:** *Tehran Heart Center, Tehran University of Medical Sciences, Tehran, Iran.*

**Keywords:** *Mitral valve*, *Heart septum*, *Heart atria*, *Atrial fibrillation*

## Abstract

**Background:** Many patients with mitral valve diseases need surgical procedures for the repair or replacement of their mitral valve. There is a great deal of controversy over the outcomes of the transseptal (TS) and left atrial (LA) approaches to the mitral valve. We sought to evaluate the outcomes of each approach more accurately by eliminating the possible biases in case selection and matching.

**Methods:** This retrospective study included patients who had surgery for mitral valve diseases via either the TS approach or the LA approach between 2004 and 2011 in Tehran Heart Center. Patients with surgical approaches other than the TS and LA were excluded. To control for the confounding effects, a propensity score matching technique was applied and the patients were matched for 14 demographic and preoperative variables. After the selection of controls, the effect of the TS approach (163 patients) versus the LA approach (652 patients) on the outcomes was presented through odds ratio (OR) with 95% confidence intervals (CI).

**Results:** The mean age of the patients was 53.15 ± 12.02 years in the TS group and 52.93 ± 13.56 years in the LA group. Females comprised 119 (73.0%) patients in the TS group and 462 (70.9%) in the LA group. There was a significant association in the prevalence of new postoperative atrial fibrillation in the two groups (OR = 1.539, 95%CI: 1.072-2.210; p value = 0.019). Temporary pacemaker placement had no statistically significant difference between the two groups (p value = 0.418). The TS patients had significantly longer pump (p value < 0.001) and cross-clamp (p value < 0.001) times. The mortality rate was 4.1% (27 patients) in the LA group and 6.1% (10 patients) in the TS group (p value = 0.274).

**Conclusion:** In our study population, the TS approach was associated with higher pump and cross-clamp times as well as risk of postoperative atrial fibrillation, but it did not increase the rates of permanent pacemaker placement, re-operations, and mortality.

## Introduction

Mitral valve diseases are among the most prevalent valvular heart diseases^1^ and necessitate surgical procedures for the repair or replacement of this valve. Conventional left atriotomy is the standard approach for most surgeons. However, the transseptal (TS) approach can confer better exposure to the mitral valve in cases where the left atrium (LA) is small, where there are adhesions caused by previous procedures, where there are concomitant operations requiring right atriotomy, and where there is beating heart surgery.^2^^-^^6^

Nevertheless, for all the advantages that the TS approach offers, controversy abounds regarding its outcome. Indeed, whereas some articles have shown that the TS approach increases the risk of postoperative sinus nodal dysfunction and atrial fibrillation, others have implicated similar and comparable results for both LA and TS approaches.^2^^, ^^7^ The question, therefore, arises as to which of these two techniques should be deemed superior. 

The major drawback of most of the studies conducted hitherto on the subject is their failure to eliminate the possible biases through proper patient selection and matching. We matched our patients with respect to 14 demographic and preoperative variables by drawing on the propensity score matching technique and studied two groups of patients with relatively similar underlying variables and achieved more reliable results.

## Methods

The present retrospective study included patients who had surgery for mitral valve diseases, between 2004 and 2011, via either the superior TS approach or the LA approach in Tehran Heart Center, affiliated to Tehran University of Medical Sciences, Tehran, Iran. The demographic data of the study population, viz. age and gender, as well as body mass index, New York Heart Association (NYHA) functional class, heart rhythm, presence of permanent pacemakers, ejection fraction, type of surgery, need for redo operations, and LA size were obtained from the Data Bank of Tehran Heart Center and matched for the patients in each of the two groups. 

In the TS approach, the right atrium was opened and a longitudinal incision (approximately 4 cm) was made in the middle of the foramen ovalis on the intra-atrial septum. The septal edges were thereafter pulled in order to expose the mitral valve fully. 

The TS and LA approaches were compared by collecting and subsequently analyzing the mean perfusion time, mean cross-clamp time, need for a temporary or permanent pacemaker after surgery, postoperative cardiac rhythm, reoperation (due to bleeding or valve dysfunction), re-sternotomy (for any reason), need for transfusion, and incidence of postoperative complications such as myocardial infarction, cerebrovascular accident, renal failure, respiratory failure, sternum infection, pneumonia, and early mortality. 

The patients who underwent surgery via approaches other than the TS and LA were excluded from the present study.

A propensity score matching technique was applied to control for the confounding effects. This technique lessens selection bias and potential confounders and enables non-random allocation in each group. A propensity score for the TS technique was performed using multiple logistic regressions. The score obtained denoted the probability of belonging in the TS group. The variables of age, gender, body mass index, heart rhythm, preoperative atrial fibrillation, ejection fraction, concomitant coronary artery bypass graft surgery (CABG) or valve surgery, previous valve operations, and baseline echocardiographic characteristics (i.e. aortic stenosis, mitral stenosis, aortic insufficiency, mitral valve regurgitation, tricuspid valve regurgitation, and pulmonary insufficiency) were included in the propensity score. These variables were selected to deactivate their effects on the postoperative outcomes. The propensity scores were used to match each patient in the TS group to 4 control patients in the LA group through a Statistical Analysis System (SAS) macro (SAS Institute Inc., Cary, NC, USA). This matching procedure initially selects matched pairs identical to 8 decimal places of the propensity scor.^8^ If no matches were found in 8 decimal places, we considered matched pairs at 7 decimal places, and so on. After the selection of controls, the effect of the TS approach versus the LA approach on the outcomes was presented through odds ratio (OR) with 95% confidence intervals (CI).

The continuous variables were presented with mean ± standard deviation (SD) or median with 1st and 3rd quartiles and were compared between the TS and LA groups using the Student t-test or the Mann-Whitney U test whenever the data were skewed and did not appear to have normal distributions. The categorical variables were described by frequency and percentage and were compared between the two groups using the chi-square test or the Fisher exact test.

## Results

Between 2002 and 2012 in Tehran Heart Center, mitral valve operations were performed on 5687 patients. The TS approach was adopted in 163 of these patients. The baseline characteristics of these patients were reviewed and matched with 652 patients, who were operated on by the same team of surgeons applying the conventional LA approach. The baseline characteristics of the study population are depicted in Table 1. A history of previous cardiac operations or other concomitant cardiac operations (CABG or valve) was not significantly different between the two groups (Table 1). There were no statistically significant differences in the baseline echocardiographic characteristics as the two groups had been matched in terms of these variables (Table 2).

**Table 1:** Baseline characteristics of the patients*

	Left Atrial Group (n=652)	Transseptal Group (n=163)	P Value
Age (y)	52.93±13.56	53.15±12.02	0.820
BMI (kg/m^2^)	25.11±4.66	25.34±4.61	0.562
Female gender	462 (70.9)	119 (73.0)	0.588
AF	223 (34.2)	54 (33.1)	0.796
Previous cardiac operations			
Valve	88 (13.5)	26 (16.0)	0.419
CABG	1 (0.2)	2 (1.2)	0.104
Concomitant valve operations	209 (32.1)	46 (28.2)	0.395
Concomitant CABG	100 (15.3)	25 (15.3)	0.999

BMI, Body mass index; AF, Atrial fibrillation; CABG, Coronary artery bypass graft

* Data are presented as n (%) or mean±SD.

**Table 2:** Baseline echocardiographic characteristics of the patients*

	Left Atrial Group (n=652)	Transseptal Group (n=163)	P Value
EF (%)	48.71±8.25	49.6±7.45	0.397
MS			0.759
Mild	9 (1.4)	2 (1.2)	
Moderate	58 (8.9)	13 (8.0)	
Severe	187 (28.7)	41 (25.2)	
AS			0.348
Mild	35 (5.4)	7 (4.3)	
Moderate	29 (4.4)	4 (2.5)	
Severe	44 (6.7)	7 (4.3)	
TS			0.479
Mild	4 (0.6)	2 (1.2)	
Moderate	10 (1.5)	2 (1.2)	
Severe	6 (0.9)	3 (1.8)	
MR			0.232
Mild	183 (28.1)	35 (21.5)	
Moderate	153 (23.5)	47 (28.8)	
Severe	217 (33.3)	52 (31.9)	
TR			0.614
Mild	35 (5.4)	13 (8.0)	
Moderate	262 (40.2)	62 (38.0)	
Severe	277 (42.5)	67 (41.1)	
PI			0.764
Mild	120 (18.4)	26 (16.0)	
Moderate	20 (3.1)	5 (3.1)	
Severe	0	0	
AI			0.055
Mild	216 (33.1)	62 (38.0)	
Moderate	118 (18.1)	17 (10.4)	
Severe	83 (12.7)	16 (9.8)	
LA enlargement			0.970
Mild	77 (11.8)	19 (11.7)	
Moderate	139 (21.3)	33 (20.2)	
Severe	371 (56.9)	96 (58.9)	

EF, Ejection fraction; AS, Aortic valve stenosis; MS, Mitral valve stenosis; MR, Mitral valve regurgitation; TR, Tricuspid valve regurgitation; TS, Tricuspid valve stenosis; PI, Pulmonary valve insufficiency; AI, Aortic valve insufficiency, LA, Left atrium

* Data are presented as n (%) or mean±SD.

**Table 3:** Patients’ postoperative outcomes and complications*

	Left Atrial Group (n=652)	Transseptal Group (n=163)	P Value
AF	179 (27.5)	60 (36.8)	0.019
Temporary pacemaker	34 (5.2)	6 (3.7)	0.418
ReOp			
Tamponade	55 (8.4)	14 (8.6)	0.952
Valve dysfunction	7 (1.1)	2 (1.2)	0.999
ICU blood transfusion	172 (26.4)	31 (19.0)	0.052
Pericardial effusion	11 (1.7)	2 (1.2)	0.999
CVA	3 (0.5)	1 (0.6)	0.999
Acute renal failure	15 (2.3)	8 (4.9)	0.107
Mediastinitis	6 (0.9)	0	0.606
Septicemia	10 (1.5)	1 (0.6)	0.703
Prolonged ventilation	53 (8.1)	20 (12.3)	0.098
Pneumonia or atelectasis	27 (4.1)	9 (5.5)	0.443
Pleural effusion	19 (2.9)	4 (2.5)	0.999
Mortality	27 (4.1)	10 (6.1)	0.274
Median pump time (min)			
In all patients	107 (80-150)	160 (120-210)	< 0.001
Pure mitral valve	92.5 (68-125.5)	150 (109.75-202)	< 0.001
Median cross-clamp time (min)			
In all patients	61 (50-90)	90 (73-141)	< 0.001
Pure mitral valve	58 (41-82)	84 (68-136)	< 0.001

AF, Atrial fibrillation; ReOp, Repeated operation; ICU, Intensive care unit; CVA, Cerebrovascular accident

* Data are presented as n (%) or median (interquartile range)

The two groups had a statistically significant prevalence of new postoperative atrial fibrillation. (OR = 1.539, 95%CI: 1.072-2.210; p value = 0.019). Temporary pacemaker placement had no statistically significant difference between the two groups (p value = 0.418). Two patients in the LA group and one in the TS group needed a permanent pacemaker. The number of re-operations for cardiac and non-cardiac causes was not significantly different between the two study groups (Table 3). Postoperative complications such as pericardial effusion, mediastinitis, septicemia, and pleural effusion were more common in the LA patients, while cerebrovascular accident, renal failure, prolonged ventilation, and pneumonia were more prevalent in the TS patients; the differences, however, did not constitute statistical significance (Table 3). One patient in the TS group had transient ischemic attack in the early postoperative period. Moreover, the need for Intensive Care Unit blood transfusion was lower in the TS patients (OR = 0.655, 95%CI: 0.427-1.006; p value = 0.053). Furthermore, the TS patients had significantly longer pump (p value < 0.001) and cross-clamp (p value < 0.001) times. 

The patients with concomitant CABG and/or valve surgery were excluded, and then the data were reanalyzed regarding the pump and cross-clamp times: the results were statistically significant (p value < 0.001).

The mortality rate was 4.1% (27 patients) in the LA group and 6.1% (10 patients) in the TS group (p value = 0.274). The associations between the surgical data and the postoperative outcomes and complications are listed in Table 4.

**Table 4:** Association between the surgical techniques and the postoperative outcomes and complications (logistic regression model)

	Odds Ratio*	95% Confidence Interval	P Value
AF	1.539	1.072-2.210	0.019
ICU blood transfusion	0.655	0.427-1.006	0.053
Renal failure	1.581	0.916-2.728	0.136
Prolonged ventilation	2.192	0.913-5.262	0.795
Mortality	1.513	0.717-3.193	0.274

AF, Atrial fibrillation

* Odds of each event in the transseptal group versus the left atrial group

## Discussion

In this retrospective study, we sought to compare the outcomes of two common approaches to mitral valve diseases: the TA and LA incisions. There have been concerns regarding the higher prevalence of arrhythmias and sinoatrial nodal ischemia in the TA approach. We evaluated the two approaches performed by a single surgeon in 815 patients and found no significant differences in the rate and rhythm disturbances between the two groups of patients. 

We succeeded in matching the baseline and preoperative characteristics of the patients in the two groups and designing a study with the fewest confounding factors. 

Our results showed that the postoperative atrial fibrillation was still higher in the TS group, even after the two groups were matched as regards the preoperative atrial fibrillation and LA size factors. One explanation for this finding may be the vulnerability of the sinus nodal artery in the TS approach, with the resultant dysfunction of the sinus node.^8^^, ^^9^ Nienaber et al.^10^ compared the mini TS approach and the LA approach and found no increase in the incidence of postoperative atrial fibrillation in the former technique. Perhaps the differences between our results and those reported by the Nienaber study are due to the shorter atrial incision, faster atrial closure time, and lesser injury to the sinus nodal artery in the mini TS approach. In this regard, Suzuki et al.^11^ introduced a new technique for the dissection and preservation of the sinus nodal artery. 

We found that the TS approach did not increase the rate of pacemaker placement. Lukac et al.^7^ reported that the TS approach was an independent risk factor for pacemaker implantation in their multivariate analysis (hazard ratio [HR]: 2.2, 95%CI: 1.2-4.1; p value = 0.014). Some other studies have shown that most post-TS approach arrhythmias are temporary and this approach does not increase the risk of permanent pacemaker implantation.^9^^, ^^12^^, ^^13^ Although a large number of our patients needed temporary pacemakers, permanent pacemakers were implanted in only 2 patients in the TS group and one patient in the LA group and the difference was not statistically significant. The TS approach did not increase re-operations due to tamponade or valvular dysfunction; this finding chimes in with the results of a study by Berreklouw et al.^14^

In the present study, the pump and cross-clamp times were longer in the TS group. Some other studies have also reported longer pump and cross-clamp times in the TS approach and considered this as an explanation for the increased rate of postoperative atrial fibrillation.^9^^, ^^10^

The rates of re-operations and mortality were not statistically different between our two study groups. 

And finally, the TS approach decreased the odds of need to Intensive Care Unit blood transfusion but increased the odds of acute renal failure in our study population. These two findings were not statistically significant. 

## Conclusion

Our results demonstrated that the TS approach was associated with higher pump and cross-clamp times and risk of postoperative atrial fibrillation but did not increase the rate of permanent pacemaker placement, re-operations, and mortality.

The TS approach is still a valuable approach to mitral valve diseases, especially in patients with a small LA and combined tricuspid and mitral valve operations. The TS approach also enables the surgeon to retract the lateral lid of the septum with a few traction sutures; consequently, this approach is a good alternative when performing the operation with limited surgical assistance.

## Notes:

***This paper should be cited as:*** Rezahosseini O, Rezaei M, Ahmadi Tafti SH, Jalali A, Bina P, Ghiasi A, Karimi A, Abbasi K, Shirzad M, Davoodi S, Salehi Omran A. Transseptal Approach versus Left Atrial Approach to Mitral Valve: A Propensity Score Matching Study. J Teh Univ Heart Ctr 2015;10(4):188-193.

## References

[1] Iung B, Vahanian A (2011). Epidemiology of valvular heart disease in the adult. Nat Rev Cardiol.

[2] Nienaber JJ, Glower DD (2006). Minitransseptal versus left atrial approach to the mitral valve: a comparison of outcomes. Ann Thorac Surg.

[3] Balasundaram SG, Duran C (1990). Surgical approaches to the mitral valve. J Card Surg.

[4] el Saegh MM, Aly MA, el Sayegh T, Mostafa EA, Zaghloul T, Saber W (1993). Transseptal approach for mitral valve surgery. A safe alternative when the need calls. Tex Heart Inst J.

[5] Aykut K, Celik B, Acikel U (2011). The transseptal approach to the mitral valve during multivalvular surgery. J Card Surg.

[6] Salerno TA, Suarez MR, Panos AL, Macedo FI, Alba J, Brown M, Ricci M (2009). Efficacy, feasibility, and pitfalls of transseptal approach in beating-heart mitral valve surgery. J Card Surg.

[7] Lukac P, Hjortdal VE, Pedersen AK, Mortensen PT, Jensen HK, Hansen PS (2007). Superior transseptal approach to mitral valve is associated with a higher need for pacemaker implantation than the left atrial approach. Ann Thorac Surg.

[8] Drago F, Turchetta A, Calzolari A, Giannico S, Marianeschi S, Di Donato R, Di Carlo D, Ragonese P, Marcelletti C (1992). Early identification of patients at risk for sinus node dysfunction after Mustard operation. Int J Cardiol.

[9] Masiello P, Triumbari F, Leone R, Itri F, Del Negro G, Di Benedetto G (1999). Extended vertical transseptal approach versus conventional left atriotomy for mitral valve surgery. J Heart Valve Dis.

[10] Nienaber JJ, Glower DD (2006). Minitransseptal versus left atrial approach to the mitral valve: a comparison of outcomes. Ann Thorac Surg.

[11] Suzuki R, Watanabe T, Matsukawa M, Hiroshige K, Sata S, Koyanagi T (2011). Sinus node artery-preserving superior transseptal approach: a simple technique. Ann Thorac Cardiovasc Surg.

[12] Misawa Y, Saito T, Konishi H (2007). The need for pacemaker implantation after using a superior transseptal approach: letter 1. Ann Thorac Surg.

[13] Tenpaku H, Wariishi S, Kanemitsu N, Okabe M, Nakamura T (2000). Combined superior-transseptal approach versus conventional approach for mitral valve surgery. Jpn J Thorac Cardiovasc Surg.

[14] Berreklouw E, Ercan H, Schönberger JP (1991). Combined superior-transseptal approach to the left atrium. Ann Thorac Surg.

